# Molecular Research on Mitochondrial Dysfunction

**DOI:** 10.3390/ijms23126845

**Published:** 2022-06-20

**Authors:** Carlo Viscomi, Maria Eugenia Soriano

**Affiliations:** 1 Department of Biomedical Sciences, University of Padova, 35121 Padova, Italy; carlo.viscomi@unipd.it; 2 Department of Biology, University of Padova, 35121 Padova, Italy

This Special Issue collects current knowledge on the molecular mechanisms underlying mitochondrial dysfunction and its related diseases, as well as therapies and perspectives pertaining to their treatment. Mitochondrial dysfunction can be the primary cause or a secondary effect of many human disorders, including neurodegenerative diseases, obesity and cancer. However, mitochondrial diseases specifically refer to a group of heterogeneous disorders characterized by impaired oxidative phosphorylation, the process by which cells transform nutrient-derived energy into ATP [1]. Mutations in both the nuclear and mitochondrial genomes can lead to mitochondrial dysfunction, and a growing body of evidence suggests that the crosstalk between the two genomes coordinates both mitochondrial and cellular functionality. In the case of mitochondria-to-nucleus retrograde signaling, the dysfunctional organelle induces alterations in the expression of nuclear genes that look to preserve functional mitochondria while eliminating defective ones [2]. Mitochondrial DNA (mtDNA)—a circular molecule of about 16.5 Kb in mammals, which is present in multiple copies—encodes 13 core proteins of complexes I, III, IV and V of the respiratory chain, as well as 22 tRNAs and 2 rRNAs required for their translation inside the mitochondrion. All the other proteins required for mtDNA replication and maintenance are encoded in the nuclear genome and actively imported into the mitochondria [1]. Due to the highly oxidative environment and the very few non-coding regions, mtDNA mutations are frequent and usually affect coding sequences; this leads to a recessive-like condition termed heteroplasmy, characterized by a mixture of wild-type and mutated mtDNA [3]. Nevertheless, the polyploid state guarantees the synthesis of enough functional mtDNA-encoded polypeptides, tRNA or rRNA over the mutated ones. The permissive threshold and physiopathological relevance are discussed in this Special Issue by Perez-Amado and colleagues. They review how heteroplasmy might evolve towards a predominant wild-type or mutated genotype in the cell, reporting mtDNA mutations that prevail in specific tumors with respect to the non-tumoral tissue, and suggest a positive contribution to the proliferation and survival of neoplastic cells [4] (Figure 1). Furthermore, Ramòn and colleagues discuss the processes and proteins governing mtDNA copy number and stability, in which dysregulation triggers the onset of mtDNA depletion and deletion syndromes [5]. 

Another fascinating topic is that of why and how mtDNA is released from mitochondria. The presence of circulating mtDNA in the plasma of patients with different pathological conditions was initially considered a technical problem during blood withdrawal and processing. However, a few years ago, several groups described an inflammatory response triggered by mtDNA release or mtRNAs during apoptosis [6,7]. In the cytosol, the mtDNA molecule triggers a pro-inflammatory and type-I interferon response by activating multiple sensor pathways. These aspects are herein reviewed by Luna-Sanchez and colleagues. The authors also discuss how aberrant innate immune signaling might contribute to the development of neurodegenerative disorders such as Huntington’s disease and Amyotrophic lateral sclerosis, or whether mt-nucleic acid release can be considered an age-related physiological marker [8]. To identify the molecular events regulating mtDNA release, it is necessary to better understand how mtDNA is organized in nucleoids, how nucleoid stability is controlled, and whether mtDNA and mtRNA release are independent or linked. Although mtDNA replication, transcription and translation occur contextually, microscopy and biochemical approaches revealed the presence of nucleic acid–protein aggregates that likely host mtDNA- or mtRNA-related processes [9,10,11]. In this Special Issue, Xavier and colleagues discuss the different putative non-membranous compartmentalization of mtDNA- and mtRNA-containing granules and their protein composition [12]. 

A limiting factor in developing new therapies for mitochondrial diseases has been the substantial lack and/or inappropriateness of suitable models. Mouse models have been, and still are, central in this process; however, they often fail to recapitulate the clinical features of human syndromes, even in the presence of the biochemical and molecular hallmarks. However, the introduction of induced pluripotent stem cells (iPSCs) has changed this paradigm, as discussed by McKnight and colleagues [13]. Interestingly, the authors also underscore some limitations in the use of iPSCs, including the challenges related to their maintenance and differentiation, and the intrinsic difficulties in using differentiated cells for drug-screening.

Despite the many difficulties in developing therapies for mitochondrial diseases, some important milestones have been reached, as discussed by Ramòn and colleagues [5]. These authors focused on mtDNA maintenance defects; these are a highly relevant group of mitochondrial diseases resulting from mutations in genes that encode components of the replication/transcription machinery, of nucleotide metabolism, and of mitochondrial dynamics. Several approaches have been tested over the last 10 years, including direct scavenging of toxic metabolites, enzyme-replacement therapy, hematopoietic stem-cell transplantation, liver transplantation, the administration of deoxyribonucleotides, and gene therapy. Additionally, metabolic reprogramming may be exploited to improve mitochondrial functions in some cancers by exploiting melatonin, an endogenous compound that is able to shift the metabolism from glycolysis to OXPHOS, as discussed by Reiter and colleagues [14]. 

Although research on the topic is still in its infancy, the possibility of having a cure for at least some mitochondrial diseases and diseases with mitochondrial alterations finally seems to be looming.

## Figures and Tables

**Figure 1:**
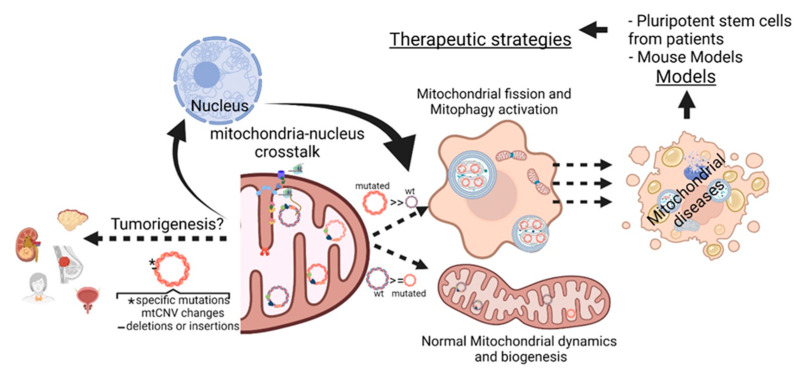
Illustration representing the different aspects discussed in this Special Issue. (created using BioRender.com, accessed on 1 September 2020).
